# On the Nature and Objects of Medical Science, and Its Relations to the Welfare of the Community

**Published:** 1872-06-01

**Authors:** N. S. Davis

**Affiliations:** Chicago, Ill.


### Original Communications.

### ON THE NATURE AND OBJECTS OF MEDICAL SCIENCE, AND ITS RELATIONS TO THE WELFARE OF THE COMMUNITY.

AN ADDRESS BEFORE THE NORTH-EASTERN INDIANA MEDICAL ASSOCIATION, JUNE 4th, 1872.

BY N. S. DAVIS, M.D., OF CHICAGO, ILL.

*Brethren of the Medical Profession, and Citizens:* I appear before you this evening to perform a task of great difficulty; namely, to so present a strictly professional subject as to interest the professional part of my audience, and at the same time make myself intelligible and instructive to the larger number who have no knowledge of either the science or the art of medicine. A desire to be relieved from pain and disease on the one hand, and a cordial sympathy with suffering, prompting efforts for its relief, on the other, are among the strongest instinctive feelings in man's nature. Hence, in the earliest records of our race, whether written or traditional, we find evidences both of human suffering and of human efforts for its relief. Direct efforts to prevent, ameliorate, and cure diseases, constitute the art or practice of medicine, and have existed in some degree among all the tribes and nations of the earth since the fall of man from his state of primeval innocence, to the present time. The science of medicine, properly so called, embraces an actual knowledge of man, and of all those surrounding agencies and influences capable of so acting as to modify his condition in health and in disease, and is of comparatively modern origin. By a knowledge of man, is meant a full comprehension of the structure and functions of every part of the living human body. To gain such knowledge requires a complete anatomical analysis, by which every bone, cartilage, ligament, tendon, muscle, nerve, blood-vessel, gland, etc., shall be studied, both separately and in their relations to each other, as well as in regard to the special function or office that each performs, as a part of the complex whole. And when the knife of the anatomist has exhausted its power of further analysis, the crucible and test glass of the organic chemist, and the microscope, take up the work and soon complete the process of opening to our view both the composition and the minute structural arrangement of every tissue entering into the organization of the human body. By this careful and extended analytical study, the human body is found to be composed of a few elementary tissues, endowed with certain properties, and each fulfilling a certain office, or performing a certain specific function peculiar to itself. It is by the varied combination of these few elementary forms of structure (no more than five in number), that all the beautifully complex and important organs of the body are formed, and made capable of acting, each in harmony with all the rest.

The five primary structures, namely, nervous, muscular, capillary vascular, secretory, and fibrous, are found in varying proportions in almost every part of the living body; and while all possess the common properties of *susceptibility*, or a capacity to receive impressions, and a *vital affinity*, by which the primary atoms or cells, of which they are made, are held in their proper places, each contributes its own specific function or peculiar action to the common object of enabling the more complex organ to perform its office. For instance, the capillary vascular structure is everywhere, both the reservoir of materials (the blood) for supplying the constant waste or change of atoms in all the structures, and the primary receptacle of the products of such waste or change. The secretory structure constitutes the active chemical laboratory by which elements are combined into new fluids, some to aid in digesting and assimilating the raw material received as food and drink; and some to be eliminated or cast out as useless or hurtful to the organization. The muscular structure furnishes the capacity for active motion; the nervous imparts sensibility, and at the same time the medium for transmitting the sensations to the proper centers; while the elastic fibrous structure affords the matrix and connecting medium by which all the other structures are held in proper position; firm enough to maintain form and durability, yet elastic enough to admit of the greatest freedom of motion. Thus, in strict accordance with the general economy of Nature, we find this wonderful and mysterious mechanism which we call the human body, composed of a small number of elements, endowed with a still smaller number of distinctive properties, but so combined as to be productive of the most varied and complicated results. Passing from the study of elementary structures to that of the fully formed and complex organs, we readily see that they are capable of being arranged into four groups, having as many distinct and important ends to accomplish. The first group embraces all the organs engaged in the reception, digestion, and assimilation of food, their end or object being to supply new material for growth and repair. The second, those that separate from the blood the waste material of the tissues, and convey it out of the system, as the skin, lungs, kidneys, etc. The third, are the seat and instruments of mind or intelligence, as the brain and its appendages, the organs of special sense, and the voluntary muscles; while the fourth group are designed for reproduction. The first may be termed organs of supply or nutrition; the second, organs of waste or excretion; the third, organs of intelligence; and the fourth, organs for the propagation of the species.

But while each group has its definite end to accomplish, and every part of each group is admirably adapted to perform its own special part of the general process, yet all are so connected, both by nervous cords and a common fluid (the blood), that they constitute links of a single chain, no one of which can be marred or broken without producing more or less disturbance throughout the whole. From this hasty glance at man's physical organization, it is seen that with the aid of the dissecting knife, the microscope, and the chemical laboratory, he stands before the truly intelligent physician of the present day, with the intricate structure and function of each tissue and organ, and their mutual influence on each other, as clearly revealed as are the lineaments of his own face when reflected from the mirror. Seeing thus clearly the natural composition, organization, or structural arrangement, and functional action of each part of the human body, and the influence that each exerts on every other, the physician has in his mind a definite standard of health; and he is prepared to appreciate and detect every departure from that standard, constituting disease. He recognizes the fact that each property common to all the tissues, and each function or action performed by the individual organs, are capable of being increased above the natural standard, depressed below it, or perverted from its proper direction, each constituting elementary forms of disease. He is thus prepared to detect at the threshold the fallacious and unphilosophical character of all those attempts to reduce all morbid action or disease to one general law. It matters not whether such law be expressed by the word *irritation* on the one hand, or *debility* on the other, or by the favorite maxim of the blunt and ignorant Samuel Thompson, that "Heat is life, and cold is death." They are equally at variance with the fact that morbid action may be in one case in the direction of irritability or excitement; in another in the direction of debility or depression; while in a third it may be in such a direction as to be incapable of being expressed by either irritation or debility, but is perverted. For instance, the action of the kidneys may be so increased as to produce a larger quantity of urine in a given time than natural, or it may be so diminished as to produce less than natural. Yet in both instances the composition of the urine may be strictly natural. But in another case, the action of the kidneys may be so perverted that the urine secreted shall contain albumen, sugar, or other unnatural ingredients, while the quantity may be strictly within the limits of health.

So, too, the action of the brain may be simply increased, producing unnatural sensibility and mental activity; or it may be diminished, causing slowness of thought, impairment of sensibility, and even paralysis; or it may be perverted, giving rise to morbid sensations and mental derangement.

If it becomes thus apparent that all attempts to reduce disease or morbid action to some one theoretical law, are futile, it follows logically that all attempts to deduce a universal law of cure, are equally unphilosophical and vain. Hence the law of universal stimulation adopted by the Brunonians of a former age, and revived in a modified form by the present generation; the opposite law of depletion or antiphlogistics; and the fanciful dogma of Hahnemann and his followers that "Like cures like," have, each in its turn, proved abortions in practice, simply because they were applicable to a limited range of actions only. Hence, whenever a so-called Doctor makes his appearance in any community, claiming to cure all diseases by some one medicine, or in accordance with some special law, it will be entirely safe to class him either as a self-deceived ignoramus, or as a designing knave, endeavoring to obtain money by false pretences.

But while a knowledge of man, such as we have hastily passed in review, constitutes an important part of medical science — so important, indeed, as to be the chief cornerstone or foundation of the temple, yet there are many other items that make up the superstructure.

Man is simply a link in the great chain of animated beings, and is constantly living and moving in the midst of agencies that are exerting an important influence on his sensitive organization. The food and drink he takes, the earth on which he treads, and the atmosphere he breathes, all exert a constant and important influence, both on the properties of his tissues and the functions of his organs. An intimate knowledge of these, therefore, becomes as much a part of medical science as a knowledge of man himself. To gain such knowledge, however, involves a careful study of the whole range of the natural sciences. The composition, geological arrangement and topography of the earth, determines the character of the emanations into the atmosphere that we must inhale every moment, as well as the quality of the water and vegetation furnished for food and drink. The composition of the atmosphere, its temperature, moisture, electrical condition, and thousand variable elements, organic and inorganic, coming, as it does, in almost direct contact with the blood in the lungs, necessarily exerts an unceasing influence, both in the promotion of health and the production of disease.

Even the food we require for sustenance, and the medicines for alleviating disease, are derived from the three great kingdoms of nature — the inorganic, the vegetable, and the animal.

Indeed, there is not a branch of either natural, physical, or mental learning which does not contribute something of importance to the superstructure of medical science. Even the instruments we use in exploring the organs for the diagnosis of disease, are exclusively the results of the application of the laws of mechanics and natural philosophy.

I have thus glanced, as rapidly as possible, at the nature and origin of legitimate medical science, for the purpose of impressing on your minds the important truth that the medical science of to-day is but a part of the great field of general science; to be acquired by the same patient study, the same mental discipline, and the same inductive processes of reasoning that we require for the successful study of any other department of human knowlege. It is not a mysterious something, that comes by intuition or hereditary descent, either to the seventh son of a seventh son, or to any reckless ignoramus, male or female, who chooses to assume the title of Doctor, and dabble with human health and life.

In truth, the science of medicine is but the selection and aggregation of such facts and principles from all the departments of human knowledge, as are calculated to aid us in understanding the structure and functions of the human system, with all the varied influences capable of affecting them, either in health or disease. The anatomy and physiology of man is only a part of the chemistry, anatomy and physiology of the organic world, vegetable and animal.

The materia medica, or, in other words, the materials for combating disease, is simply an aggregation of items from chemistry, botany, mineralogy, zoology, and general physics. While disease itself, instead of being a mysterious entity — a physical or imponderable something pervading the human organization, and capable of being driven about from tissue to tissue, or exorcised by charms, incantations, or the laying on of hands—is simply a deviation or departure of some structure or function from the natural standard of action, produced either by some one of the various agents acting through the air we breathe, the food and drink we take, or the mental impressions we receive, or by hereditary influence. And rational medicine consists in a clear conception, first, of the natural or healthy standard of structure and action; second, of the special direction and extent of the deviation from the natural in any given case of disease; and third, of the nature and action of such remedial agents as are capable of counteracting the unnatural deviation and aiding in the removal of its effects. The skill and success of every physician will depend on the extent and accuracy of his knowledge in these three particulars, and on the promptness and precision with which he selects and adapts the proper remedies to the nature and stage of progress in each individual case of disease. If he finds the deviation from healthy action to be in the direction of excess or excitement, he applies agents of a soothing and sedative nature; if in the direction of depression or debility, he aids the restoration by tonics; or, if in the direction of perverted action, he applies neutralizers and alteratives. In other words, he follows the ordinary teachings of common sense and rational induction, by soothing pain, repressing excitement, propping up debility, and correcting perverted action; instead of constructing a theoretical iron bedstead, in the form of some pretended panacea, therapeutic law, or special *pathy*, or *ism*, to which all patients and all morbid actions are to be fitted, *nolens volens;* whether such *bedstead* be represented by the shocks and baths of Electropathy; the duckings and packings of Hydropathy; the infinitesimal sugar pellets selected by the rule of "*Similia similibus*" of Homœopathy; or the half dozen roots and herbs of the more vulgar, though not more absurd, "root-doctor."

The true physician is the enlightened handmaid and helper of nature, in the broadest and truest sense. The great object of his life is to comprehend clearly the deviations, the perversions, and obstructions, to which the various parts of the living human body are subject, and to be ever ready to bring some agent or influence to bear for counteracting the deviations and removing the obstructions. He does not play the part that some, eminent in our profession, and even occupying high positions, would fain assign to him. They not only exalt *nature* to something possessing the attributes of intelligent personality, if not Deity, but they also *personify* disease; and through all their writings runs the clearly expressed idea that the latter is in active combat with the former; as much so, indeed, as two armies marshaled in the field of physical strife. And their favorite position for the physician to occupy, appears to be that of a faithful sentinel, with a little reserve corps at his command, by the aid of which he is to ensure fair play between the combatants; and especially to see that *nature* does not become embarrassed during the fight by bad air, injudicious or insufficient food, and neglect of cleanliness; but in no case is he to violate the laws of neutrality by active interference in the combat. That I do not exaggerate or misrepresent the ideas that have come to us from the Bigelows, Forbeses, Holmeses, and other so-called conservatives, in modern medical literature, permit me to quote the following from an address of Dr. Gibson before the British Medical Association, in 1870. He says: "Diseases have, so to speak, a lifetime of their own, with its periods of growth, maturity, and decline. They are the passing tenants of the body, which they occupy often with great injury for a limited time. Treatment cannot change their nature, cannot expel them at once, cannot quench them, cannot materially shorten or prolong their existence." Could language be chosen that would convey a more perfect idea of a living bodily entity, than we find here applied to diseases? They are not represented as mere morbid actions or perverted functions, induced by the undue impression of some exterior influence, but they are actual "tenants,"—living occupants, capable of growth, maturity, and decline. The physician may look on anxiously, and endeavor to prevent these occupants from breaking the windows, tearing down the partitions, or rooting up the foundations of the human tenement; but he can do nothing towards destroying the unwelcome intruders, or even hastening their departure.

Such doctrines are not only devoid of all foundation in the facts of science, but they have a most injurious effect on the progress of true rational medical practice. They are only the reproduction, in new phraseology, of the same ideas that were embodied by the ancients in their *vis animæ*, *vis vitæ*, *vis medicatrix naturæ*, etc. They degrade the physician to the position of a gentlemanly looker-on, with here and there a word of advice to the nurse in regard to the hygiene of the sick-room, and perhaps a placebo for effect on the mind of the patient, while the latter patiently takes his chances for life or death in his unaided struggle with the "tenants" that have taken temporary possession of his clay tenement. Will some of the toil-worn practitioners in my audience, who have for years combated disease on the rich and beautiful prairies around us, tell me what kind of bodily existence is assumed by intermittent fever when it becomes a tenant of the human body; how long it continues to grow; what the duration of its sturdy maturity; and what the rate of its decline? Are you satisfied that you have never changed his complexion, or hastened somewhat his departure by a few doses of a suitable anti-periodic? Will any of you give me the same facts in regard to bronchitis, pneumonia, dysentery, cholera morbus, etc.? That we have some diseases which, like the eruptive fevers, run a definite course, we freely admit. This is not, however, because they have a bodily existence, but simply because they are produced by specific viruses, which, when once introduced into the blood, can be eliminated only slowly, and by the aid of certain morbid processes; and for the neutralization of which we have yet discovered no reliable antidote.

True scientific and rational medicine neither consists in placidly waiting to see how large a proportion of patients will finally get well without active medication, on the one hand; nor in blindly and heroically dosing every sick person, merely because they are sick, on the other; but in studying assiduously the causes and nature of every variety of morbid action, and the accurate and prompt application of such means as will remove or neutralize the causes, and lessen the intensity and duration of the morbid actions.

Actuated by such a purpose, his practice neither becomes amenable to the charge of being a "meditation on death," nor the work of a blind man with a club, striking at random, and hitting the patient or the disease, as simple chance may dictate; but he is stimulated to constant activity in the acquisition of new facts and principles, and the more accurate adjustment and use of those already acquired. His mental vision, instead of becoming narrowed to the limits of a special dogma or ism, is constantly enlarging its scope, more clearly defining the relation of its facts, and more accurately applying them to the great work of ameliorating human suffering, and prolonging human life. He not only lays under contribution every department of human knowledge, either for facts or materials with which to prosecute his noble work, but his clear perception of the need of more facts and materials, makes him in turn one of the most active and successful cultivators of the very sciences that he has laid under contribution for the benefit of himself and his patients.

This is the reason why, in all ages, members of the medical profession have been among the most active and eminent cultivators of every department of physical and mental science.

Having glanced at the nature of medical science, and the art or practice, so far as it relates to the treatment of disease, I should come far short of doing justice, did I omit to allude to another and perhaps even nobler application of our science.

It is a great and beneficent work to alleviate suffering by lessening the intensity, shortening the duration, or curing disease; but it is a more desirable, if not nobler work, to prevent its occurrence. Hence the application of the truths of our science to the construction of hygienic and sanitary measures, by which not only individuals, but whole communities, are protected from the operation of causes that would otherwise induce annually millions of cases of disease and thousands of deaths, constitutes one of its most gratifying achievements — achievements, indeed, which constitute a broad line of distinction between legitimate medicine and all the various special systems and pathies that have existed from the days of Paracelsus, who died with his boasted Elixir of Life in his pocket, to the present time. Who can tell how many lives have been saved, and how many fair faces have been kept free from the pits of small-pox, during even the last twelve months, by the well-known discovery of Jenner? Who can tell how much human suffering has already been prevented by the discovery and application of anæsthesia by Wells, Jackson, Simpson, and their co-laborers? Or who can compute the grand total of sickness and mortality in the great cities of the world, prevented by the application of the facts and principles of medical science to the work of drainage, ventilation, disinfection, etc.? If any wish to test their mathematical skill, let them compare the ratio of mortality of London, before the great fire of 1666, with that of the London of to-day. And when they have figured up even the column of lives saved in that city alone during the last two centuries, let them apply the same estimate to all the densely-populated cities of the civilized world. And yet, what a field is here still open for extension and improvement! Indeed, all that has been accomplished in sanitary science, or preventive medicine, is only the beginning of a work now in its infancy. The further application of known facts and principles to the sanitary improvement of cities; the extension of the same principles to the selection of the site and the construction of every farm-house in the country; and the further discovery of the causes of diseases and their antidotes, both in and out of the human body, by the aid of organic chemistry and the microscope, are all open fields of professional labor, the future results of which will make the work already accomplished appear, to future generations, as only the small drops that herald the coming of the copious aud refreshing shower.

And yet, this whole work of sanitary improvement springs directly and logically from the intimate connection between legitimate medicine and the collateral sciences. It is that connection which developes clearly and fully the intimate relation of man, as a physical being, to the physical agencies that surround him; and the same inductive processes of reasoning that we have indicated as governing the application of remedies to the treatment of diseases, necessarily suggest the importance of their prevention by a removal of the causes by which they are induced. But what kind of sanitary measures for the prevention of disease have ever sprung from the study of any of the various special systems or pathies that have either blessed or cursed the human race? Where are the systems of sewerage, cleanliness, water supply, ventilation, etc., on which so much of the health and comfort of all modern densely-populated towns depend, which have been devised by the disciples of Electropathy, Hydropathy, Eclecticism, and Homœopathy? Indeed, what kind of sanitary measures would be logically suggested by the so-called great therapeutic law of Hahnemann, "*Similia similibus curantur*"? Surely not by sewers, disinfectants, and pure water, for that would be acting on the law of contraries. Would it not be by attempting to cure one foul air by bringing in contact with it another as near like it as possible? One bad odor by another closely resembling it? I have alluded to these isms and pathies, not that they are deemed of sufficient importance to elicit opposition, but simply to illustrate more clearly the true character of legitimate medicine. They have always existed in some form, and will probably continue to exist so long as there are erratic minds in the human family. Parallel excrescences exist in every profession and human calling. The honorable profession of Law has its pettifoggers and shysters ; and that of Theology has its erratic off-shoots of every grade; and all find followers. Even the infidel, who is so credulous as to believe that this world, with the evidences of intelligent design inscribed upon every rock, murmured from every brook, exhaled from every opening flower, whispered from every fiber of his own organization, and thundered from the starry sky, has no other creator than blind Chance, has plenty of followers. The true science and art of medicine has nothing to fear from such off-shoots. With its facts and principles culled from every department of general science, its progress and expansion will move on, as they have hitherto moved, *pari passu* with the advance in other departments of learning, always quickened by the instinctive desire to sieze upon every fact and influence that can be made available for the relief or prevention of human suffering.

If I have made myself understood by this intelligent audience, in regard to the nature of medical science and practice, and its intimate relations to the individual and social interests of man, I trust there is not one of you who will longer look upon the profession as a mere trade or calling by which a class of individuals gain a respectable livelihood, but you will appreciate and foster in every proper way its advancement to the more perfect accomplishment of its high and beneficent purposes. Not only smile on its votaries when they stand by your couch of pain, or with manly courage brave the pestilence while all others quail or flee, but see to it that every obstacle to the advancement of the science and the complete education of the profession is removed. By just laws provide for the legal study of human anatomy; by a wise and liberal economy encourage every intelligent effort for the sanitary improvement of communities, and by a still more liberal policy see to it that your sons who study medicine have the advantage of that thorough mental discipline and development that a liberal education alone can give. And you, brethren of the profession, falter not in the work of your great mission, but continue to mingle in your social capacities; continue to foster friendly intercourse and mutual improvement, ever remembering that the great end of life is to do good, and that he is the noblest hero who does most to leave the world better than he found it.

